# Analysis of Physiological Indicators Associated with Drought Tolerance in Wheat under Drought and Re-Watering Conditions

**DOI:** 10.3390/antiox11112266

**Published:** 2022-11-16

**Authors:** Jiarui Wang, Xiaoyan Zhang, Zhidong Han, Haoxiang Feng, Yangyang Wang, Juan Kang, Xiaojie Han, Lifang Wang, Chenyang Wang, Hua Li, Geng Ma

**Affiliations:** 1 College of Resources and Environmental Sciences, Henan Agricultural University, Zhengzhou 450046, China; 2 College of Agronomy & State Key Laboratory of Wheat and Maize Crop Science, Henan Agricultural University, Zhengzhou 450046, China; 3 National Engineering Research Center for Wheat, Henan Agricultural University, Zhengzhou 450046, China; 4 Henan Technology Innovation Center of Wheat, Henan Agricultural University, Zhengzhou 450046, China; 5 College of Life Science, Henan Agricultural University, Zhengzhou 450046, China

**Keywords:** wheat, drought, physiological characteristics, drought tolerance indicators

## Abstract

Wheat (*Triticum aestivum* L.) production is severely threatened by an increase in the frequency of drought events. It is crucial to determine stable and effective morphological, physiological, and associated oxidative stress indicators, to evaluate the drought tolerance of wheat for breeding and cultivation. Therefore, the cultivars Luohan 22 (LH 22, drought−tolerant) and Zhengmai 366 (ZM 366, drought−sensitive) were used as experimental materials to analyze the changes in 12 physiological and biochemical indicators, as well as the yield, when the stress was prolonged to different times. Re-watering after 6 days of drought can effectively alleviate the associated oxidative stress of drought to wheat. The physiological responses of plants were reversible when they were re-watered in the range of 6 to 12 days after drought. The degree of recovery of LH 22 was higher than that of ZM 366. Afterwards, seven indicators, including stomatal conductance, proline, malondialdehyde, soluble sugar, hexokinase, glucose, and the non−photochemical quenching parameter, were screened out to characterize tolerance of wheat to drought using the multivariate statistical analytical method. This study further investigated the method of evaluating and indexing tolerance of wheat to drought, from the physiological and biochemical levels. This study can provide a theoretical basis and reference for the selection of wheat cultivars to breed and cultivate against drought stress.

## 1. Introduction

Globally, the occurrence of drought hazards is gradually becoming frequent and intense under climate change [1,2]. One of the major environmental factors that limits global crop yields is drought stress, which also makes it difficult for crops to complete their life cycle [3]. Water shortage is another environmental factor that inhibits many metabolic processes, thus affecting crop survival, growth, and crop productivity [4].

The crop responds to drought stress in a variety of physiological and biochemical manners, in order to survive [5]. One of the initial responses to drought is stomatal closure [6], which can limit plant transpiration and CO_2_ uptake, resulting in a reduction in photosynthesis [7]. Stomatal limitation is considered to be the primary factor that leads to reduced photosynthesis when the effective water content in the soil is insufficient. Moreover, non−stomatal limitations, such as a decrease in ribulose-1,5−bisphosphate carboxylase-oxygenase (Rubisco) activity, CO_2_ availability in the chloroplast, and photosystem II (PSII) photochemical efficiency, take place when the intensity and duration of water stress increase [8,9]. Moreover, reactive oxygen species (ROS) that are produced in response to drought stress can trigger oxidative stress, which leads to the peroxidation of cell membrane lipids [10]. The ROS in cells can quickly be removed to maintain the stability and function of the cell membrane structure. This is done by increasing the activity of important components of the antioxidant protective enzyme system, such as superoxide dismutase (SOD), catalase (CAT), and peroxidase (POD), which are often used as physiological indicators of the stress tolerance of crops [11,12]. These enzymes can reduce the toxic effect of ROS on cells, and improve drought tolerance [13]. In addition to the rapid accumulation of glucose and fructose, drought can also lead to increased concentrations of soluble sugars and carbohydrates in leaves, and induce the expression of hexokinase (HXKs) [14].

The physiological characteristics of plant drought tolerance can not only be observed under drought stress, but throughout the recovery process after re-watering. For example, re-watering plants after a drought can immediately restore their physiological functions, increase the rate of photosynthesis, and accelerate their growth [15] by rapidly growing new tissues, reopening stomata, and reducing peroxidation. The recovery of metabolic activity is also promoted by osmotic regulation after re-watering [16]. Generally, the degree of recovery from re-watering is strongly dependent on the plant species, intensity, and duration of the drought [17,18].

The tolerance of plants to drought stress is quite complex, and is controlled by genetic, physiological, and biochemical mechanisms. The effects of drought stress vary with intensity, duration, and the phenological period. To manage the stress that is related to water scarcity, plants have evolved tolerance in a complex way, and inaccuracies arise when the drought tolerance of crops is evaluated using only one indicator [19,20]. Therefore, a combination of methods, including the drought tolerance coefficient method, principal component analysis, membership function method, grey relational analysis, and stepwise regression, was used in this study to comprehensively evaluate the crop. These methods have been widely used for studies in wheat [21,22], barley [23], maize [24], and cotton [25], for a long time. However, in previous studies, the evaluation of drought tolerance of wheat cultivars was primarily based on the morphological index, and only a few studies focused on changes at the physiological level. Changes at the physiological and biochemical levels can reflect the responses of crops to drought and the compensation effect after re-watering, in a timely manner. Since wheat is more vulnerable to drought stress during the booting stage, drought causes a significant reduction in the number of panicle grains, and subsequently decreases the yield [26]. Therefore, a drought re-watering experiment was designed at the booting stage in this study, with the aim of clarifying (a) the effects of prolonging the drought stress on physiological indicators, and the degree of recovery after re-watering; and (b) selection of the best drought tolerance indicators and associated evaluation techniques. This study should provide a reference to breed drought tolerant cultivars, as well as means of identifying wheat drought tolerance in the future.

## 2. Materials and Methods

### 2.1. Experimental Site and Growth Conditions

Pot experiments were conducted in the wheat growing season of 2018–2019 at the Science and Education Experimental farm of Henan Agricultural University, Zhengzhou, China (34.44° N, 113.42° E). The experimental site is characterized by a north temperate continental monsoon climate zone with a total duration of sunshine during the whole growth period of 1044 h. The effective accumulated temperature is 2467.7 °C. The mean relative humidity is 52.6%, and the total rainfall is 89 mm.

Tillage layer of 0–20 cm soil with a field capacity of 23.70% was used for the experiment. The relevant soil parameters were tested to evaluate the availability of nutrient elements, and identified the organic matter at 15.52 g·kg^−1^, total nitrogen at 0.78 g·kg^−1^, available phosphorus at 20.73 mg·kg^−1^, and available potassium at 254.02 mg·kg^−1^. The seeds were sown in 27 cm high and 26 cm diameter pots that contained 10 kg of loam soil that had been sieved with a 5−millimeter mesh. The pots were buried underground, which kept the soil surface within the pot at the same level as that of the field [27]. Fertilizer was applied at a rate of 2.23 g of nitrogen, 8.30 g of P_2_O_5_, and 1.78 g of K_2_O per pot before sowing on October 18, 2018. The seedlings were fixed at the three-leaf stage; there were 12 plants per pot, and a top dressing of 2.23 g of nitrogen was applied per pot at the jointing stage.

### 2.2. Experimental Design and Plant Materials

Two wheat cultivars, the drought tolerant cultivar Luohan 22 (LH 22) and the drought−sensitive cultivar Zhengmai 366 (ZM 366), were selected for the potting experiments. Before water control, all of the treatments were watered well to ensure normal plant growth. The water stress treatments were conducted at the booting stage of wheat development. Two different water treatments were applied. Well−watered (WW), 75% ± 5% of relative soil water content during the whole growth stage, was taken as the control. Drought stress (DS), 50% ± 5% of the relative soil water content, was applied for 6 d, 12 d, and 18 d (denoted as D 6, D 12, and D 18, respectively). All of the treatments were then re-watered to normal water conditions (denoted as R 6, R 12, and R 18, respectively). The water content was controlled one week in advance by a combination of the weighing method and a soil moisture analyzer (TDR 300). The experiment was conducted in rain−shelter conditions, in order to prevent the effect of natural precipitation. Flag leaves were sampled at the end of drought treatments, and at three days after re-watering, while the WW treatment was sampled at the corresponding time as the control. All of the samples were stored at −80 °C for analysis. Each treatment had 12 replicates, and a total of 216 pots were used for the experiment (2 cultivars × 3 treatments [WW + DS + R] × 3 prolonged stress × 12 replicates = 216 pots).

#### 2.2.1. Photosynthetic Gas Exchange Parameters

On clear days, a portable photosynthesis measurement system (Li−6400; LI−COR Inc., Lincoln, NE, USA) was used to measure the net photosynthetic rate (Pn) and stomatal conductance (G_s_) of the wheat flag leaves between 9:00 and 11:00. The light source was established at 1000 μ mol m^−2^ s^−1^ photosynthetic photon flux density (PPFD), and the CO_2_ concentration was 400 μ mol mol^−1^ during the measurements. The plants that had consistent growth were measured in triplicate for each treatment.

#### 2.2.2. Chlorophyll Fluorescence Parameters

Flag leaves with consistent plant growth were chosen. Three replicates for each treatment were used to measure the chlorophyll fluorescence parameters using a chlorophyll fluorometer MINI−PAM−Ⅱ (Walz, Effeltrich, Germany), including the maximum photochemical quantum yield of PSII (Fv/Fm), coefficient of photochemical quenching (qP), non−photochemical quenching parameter (NPQ), and coefficient of non−photochemical quenching (qN).

#### 2.2.3. Biochemical Parameters

Flag leaves that had consistent plant growth were chosen, and three replicates for each treatment were used to measure the activities of SOD and HXKs, as well as the contents of malondialdehyde (MDA), proline (Pro), soluble sugar (SS), and glucose (GLC), using kits provided by Suzhou Keming Biotechnology Co., Ltd. (Suzhou, China).

The activity of SOD was assayed at 560 nm on the basis of inhibition of nitroblue tetrazolium (NBT) photochemical reduction. The content of MDA was determined using the thiobarbituric acid (TBA) test. The activity of HXKs was determined spectrophotometrically. Briefly, glucose was converted to glucose-6−phosphate by hexokinase [28]. The catalytic dehydrogenation of glucose-6−phosphate was conducted and transformed into nicotinamide adenine dinucleotide phosphate (NADPH). The amount of NADPH was determined by the absorbance at 340 nm.

Wheat flag leaf tissues (0.1 g) were ground with 1 mL of extraction solution in an ice bath, followed by centrifugation for 10 min at 8000× *g* at 4 °C. The supernatant was collected, and subsequently determined according to the manufacturer’s instructions.

The contents of Pro were measured using the sulphosalicylic acid method. Wheat flag leaf tissues (0.1 g) were extracted in 1 mL of extraction solution, followed by incubation of the homogenate in a 95 °C water bath for 10 min. The homogenate was then centrifuged for 10 min at 10,000× *g* at 25 °C. The supernatant was collected, and the content was determined according to the manufacturer’s instructions.

The contents of SS were determined using the anthrone method. The amount of GlC was measured using the glucose oxidase method [29].

Wheat flag leaf tissues (0.1 g) were extracted in 1 mL of distilled water and then incubated in a 95 °C water bath for 10 min. After cooling, the homogenate was centrifuged for 10 min at 10,000× *g* at 25 °C. The supernatant was collected and subsequently determined, according to the manufacturer’s instructions.

#### 2.2.4. Grain Yield

The number of effective spikes per pot and grains per spike were recorded. After the wheat was harvested, threshed, and sun−dried, the 1000−grain weight was measured. The grain yield at corresponding moisture contents was recorded and expressed against a standard moisture content of 13%. Six pots of each treatment were reserved for yield measurements.

### 2.3. Statistical Analysis

The data were processed using Microsoft Excel 2013 (Redmond, WA, USA). The data were fitted, and figures were generated using Origin8.5 (OriginLab, Northampton, MA, USA); SPSS 19.0 (IBM, Inc., Armonk, NY, USA) was used to perform the statistical analysis. The individual drought tolerance coefficient (DC) (Equation (1)) and comprehensive drought tolerance coefficient (CDC) (Equation (2)) were calculated using the following equations:

(1) $$\mathrm{DC}=\frac{x_{i}}{ck_{i}}$$

(2) $$\mathrm{CDC}=\frac{1}{n}\overset{n}{\underset{i=1}{\sum }}\mathrm{DC}$$

where *x_i_* and *ck_i_* represent the measured values of drought stress and re-watering for the treatment and control, respectively [20].

A simple correlation analysis, statistical analysis of continuous variables number distribution, and principal component analysis were conducted to calculate the DC value of each index. The factor weight coefficient (*ω_i_*) (Equation (3)), the membership function value of each composite indicator [*μ*(*x_i_*)] (Equation (4)), and the drought tolerance comprehensive evaluation (D) (Equation (5)) were calculated using the following equations [21]:

(3) $$\omega_{i}=P_{i}÷\overset{n}{\underset{i=1}{\sum }}P_{i}$$

(4) $$\mu (x_{i})=\frac{x_{i}-x_{i,min}}{x_{i,max}-x_{i,min}}$$

(5) $$D=\overset{n}{\underset{i=1}{\sum }}\left[\mu (x_{i})\times \left(P_{i}÷\overset{n}{\underset{i=1}{\sum }}P_{i}\right)\right]\;$$

where *P_i_* is the contribution rate of the *i*−th comprehensive index, indicating the importance of the *i*−th index in all the indices. *x_i_* refers to the comprehensive index, and *x_i,__max_* and *x_i,__min_* are the maximum and minimum values of the *i*−th comprehensive index, respectively.

A grey relational analysis with the DC value of each index as the comparison series, and the D value as the reference sequence, was used to obtain the degree of correlation (*γ*D) between the DC value of each index and the D value. The weight coefficients [*ω_i_*(*γ*)] and weight drought tolerance coefficients (WDC) were calculated according to Equations (6) and (7), respectively.

(6) $$\omega_{i}=\gamma_{i}÷\overset{n}{\underset{i=1}{\sum }}\gamma_{i}$$

(7) $$\mathrm{WDC}=\overset{n}{\underset{i=1}{\sum }}\left[\mathrm{DC}\times \left(\gamma_{i}÷\overset{n}{\underset{i=1}{\sum }}\gamma_{i}\right)\right]\;$$

where γ*i* is the correlation degree of each indicator.

Taking the DC value of each index as the comparison sequence, and the WDC value as the reference sequence, a grey relational analysis was performed to obtain the correlation degree (γWDC) between the DC value of each index and the WDC value. Finally, according to the D value of each index, with the CDC value and WDC value as the reference sequence, stepwise regression analysis was conducted on the DC value of each index to obtain the regression equation [23].

## 3. Results

### 3.1. Effects of Drought Stress and Re-Watering on Yield and Phenotype

**Figure 1:**
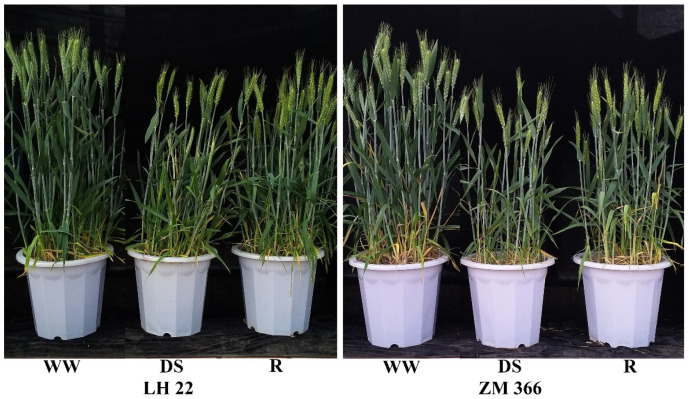
The phenotypes of LH22 (drought−tolerant cultivar) and ZM366 (drought−sensitive cultivar) under drought and re-watering. WW: Well−watered; DS: Drought stress; and R: Re-watering.

The two cultivars showed significant differences under drought re-watering conditions (Figure 1). The growth pattern of LH 22 under drought conditions was better than that of ZM 366, and there were fewer senescing leaves at the plant bottom. Both cultivars significantly recovered in growth after they were re-watered. The results from the experiment revealed that stress significantly decreased the grain yield of two different wheat cultivars. In comparison to WW, the drought stress decreased the yields of LH 22 and ZM 366 by 50.02% and 59.34%, respectively. Furthermore, it was observed that re-watering helped to reduce the loss of yield caused by drought stress. Re-watering after 6, 12, and 18 days of stress reduced the losses of LH 22 by 31.52%, 19.11%, and 4.10%, respectively, and those of ZM 366 were reduced by 30.02%, 16.73%, and 4.62%, respectively. The lowest yield loss was observed in the treatment of R 6 for both wheat cultivars, and ZM 366 was found to be more sensitive to drought stress than LH 22 (Table 1).

### 3.2. Changes in the Photosynthetic Characteristics and Chlorophyll Fluorescence Parameters under Drought Stress and Re-Watering Conditions

**Figure 2:**
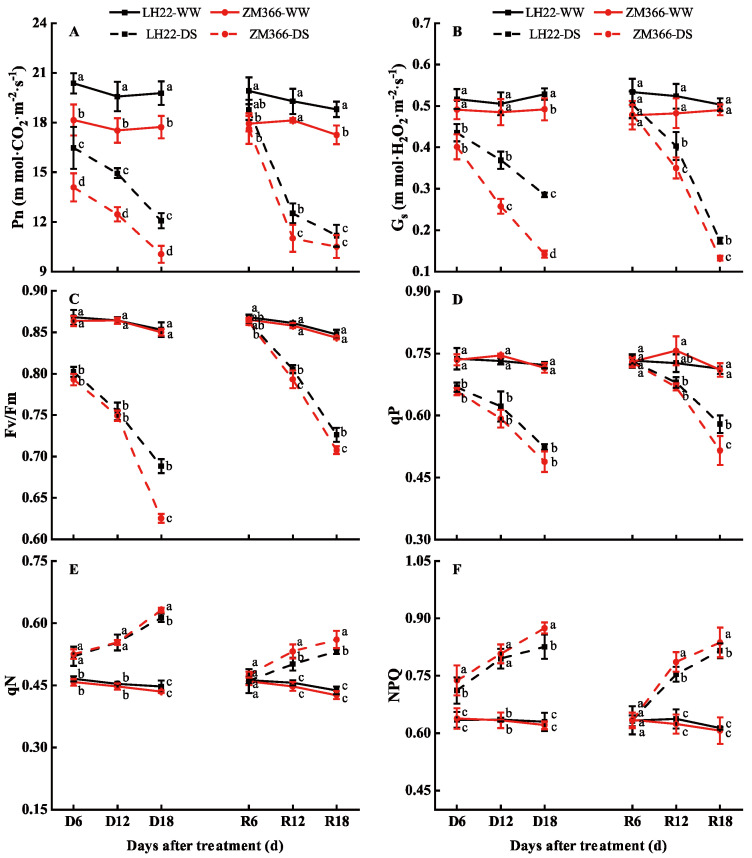
Effects of drought re-watering on (**A**) net photosynthetic rate (Pn), (**B**) stomatal conductance (G_s_), (**C**) maximum photochemical quantum yield of PSII (Fv/Fm), (**D**) coefficient of photochemical quenching (qP), (**E**) coefficient of non−photochemical quenching (qN), and (**F**) non−photochemical quenching parameter (NPQ) of flag leaves of wheat at the booting stage. WW: well−watered; DS: drought stress; D 6, D 12, and D 18 indicate 6 d, 12 d, and 18 d of stress, respectively. R 6, R 12, and R 18 indicate re-watering after 6 d, 12 d, and 18 d of stress, respectively. Different lowercase letters in the same column indicate significant differences between the treatments (LH 22−WW, ZM 366−WW, LH 22−DS, and ZM 366−DS) at *p* < 0.05 (Duncan).

Drought stress induced a continuous decrease in the Pn and G_s_ compared with those of the WW. The Pn and G_s_ decreased more in the drought−sensitive cultivar (ZM 366) than the drought−tolerant cultivar (LH 22). It was observed that re-watering after 6 days of drought restored the Pn and G_s_ to normal levels in both cultivars, but the recovery effect in terms of the Pn and G_s_ was poor when they were re-watered after 12 and 18 days of drought. The Pn decreased by 35.13% and 40.62% in LH22, and by 39.31% and 41.20% in ZM 366 at R 12 and R 18, respectively. The G_s_ was also reduced by 23.14% and 65.30% in LH 22, and by 27.41% and 73.02% in ZM 366 at R 12 and R 18, respectively (Figure 2A,B). 

Drought stress caused a significant decrease in Fv/Fm and qP (Figure 2C,D), which progressed as the time of stress was prolonged. The qN and NPQ (Figure 2E,F) showed a progressive increase as the stress was prolonged. It is obvious that the magnitude of changes (up or down) in Fv/Fm, qP, qN, and NPQ under drought stress was greater in ZM 366 than in LH 22. After re-watering, Fv/Fm, qP, qN, and NPQ were gradually restored to the WW level at R 6, but these four fluorescence parameters did not return to normal levels for both cultivars under the R 12 and R 18 treatments. Under both drought and re-watering conditions, LH 22 had better Fv/Fm and qP, while ZM 366 obtained higher values for qN and NPQ.

### 3.3. Changes in Antioxidant Enzyme Activity, Osmoregulatory Substances, and Sugar Content under Drought Stress and Re-Watering Conditions

**Figure 3:**
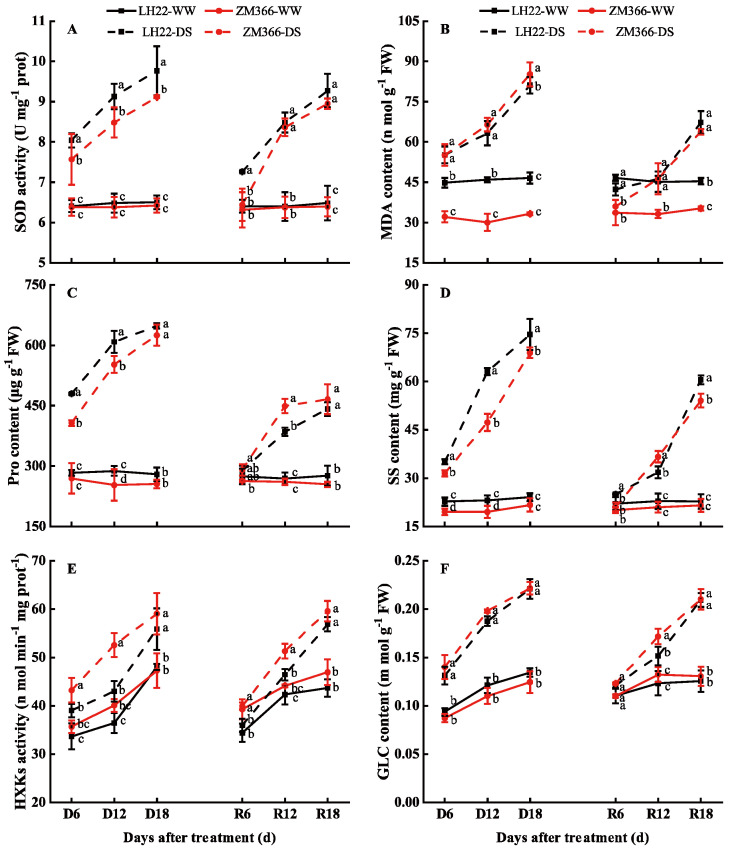
Effects of drought−re-watering on (**A**) superoxide dismutase (SOD), (**B**) malondialdehyde (MDA), (**C**) Proline (Pro), (**D**) soluble sugar (SS), (**E**) hexokinase activity (HXKs), and (**F**) glucose content (GLC) in the flag leaves of wheat at the booting stage. WW: well−watered; DS: drought stress; D 6, D 12, and D 18 indicate 6 d, 12 d, and 18 d of stress, respectively. R 6, R 12, and R 18 indicate re-watering after 6 d, 12 d, and 18 d of stress, respectively. Different lowercase letters in the same column indicate significant differences between the treatments (LH 22−WW, ZM 366−WW, LH 22−DS, and ZM 366−DS) at *p* < 0.05 (Duncan).

When compared with WW, the activity of SOD and the contents of MDA, Pro, and SS increased significantly as the time of stress was prolonged for both cultivars, but the increase in MDA content of LH 22 was lower than that in ZM 366. The values increased by 23.11%, 37.84%, and 74.32% in LH 22, while they increased by 71.50%, 121.04%, and 156.31% in ZM 366 after 6, 12, and 18 days of stress, respectively (Figure 3A–D). After re-watering, the SOD activity and the contents of MDA, Pro, and SS of both cultivars significantly decreased, and were restored to the WW level at R 6; however, only the MDA content of LH 22 was restored to the WW level at R 12.

Under the drought stress conditions and with prolonged stress, the activity of HXKs and the content of GLC in both cultivars increased gradually compared with the WW conditions. The magnitude of increase in HXKs activity was greater in ZM 366 than in LH 22 under drought stress treatment. After re-watering, the HXKs activity and GLC content in both cultivars decreased rapidly, and were restored to levels comparable to WW at R 6; however, as the stress was prolonged, the functional leaves were damaged and failed to be restored to the WW level. Compared with WW, the HXKs activity still increased by 12.03% and 44.51% in LH 22, and by 29.04% and 45.90% in ZM 366 at R 12 and R 18, respectively. The content of GLC increased by 23.11% and 66.52% in LH 22, and by 29.71% and 61.04% in ZM 366 at R 12 and R 18, respectively (Figure 3E,F).

### 3.4. Analysis of Variance

A combined analysis of variance (ANOVA) showed that all of the physiological indicators were found to be significant under varying amounts of soil moisture and duration of drought stress. With the exception of Pn, SOD, and SS, significant differences were found in all of the other indicators for both cultivars. There were significant differences in SS and Fv/Fm in a tripartite M × C × SD interaction. The Pn, G_s_, MDA, GLC, and Fv/Fm reached a significant level in the M × C interaction, and there were also significant interaction effects in Pn, G_s_, Fv/Fm, Pro, SS, GLC, and HXKs between the M × SD interaction, while a significant interaction effect only existed in the G_s_, MDA, and Fv/Fm between the C and SD (Table 2). This indicated that the duration of drought stress had significant differences in the most important parameters followed by soil moisture and cultivar.

### 3.5. Comprehensive Evaluation of Wheat Drought Tolerance

#### 3.5.1. Drought Tolerance Coefficient of Individual Indicators and Correlation Analysis

**Figure 4:**
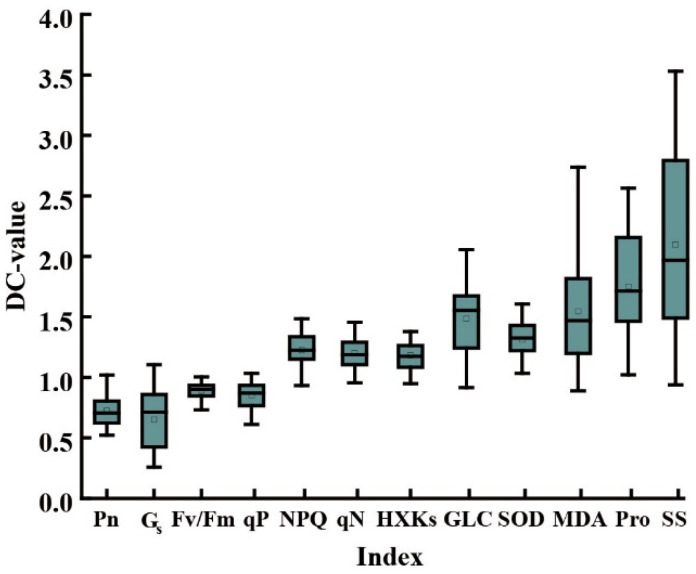
Box plot showing variation in the drought tolerance coefficient for each index.

**Figure 5:**
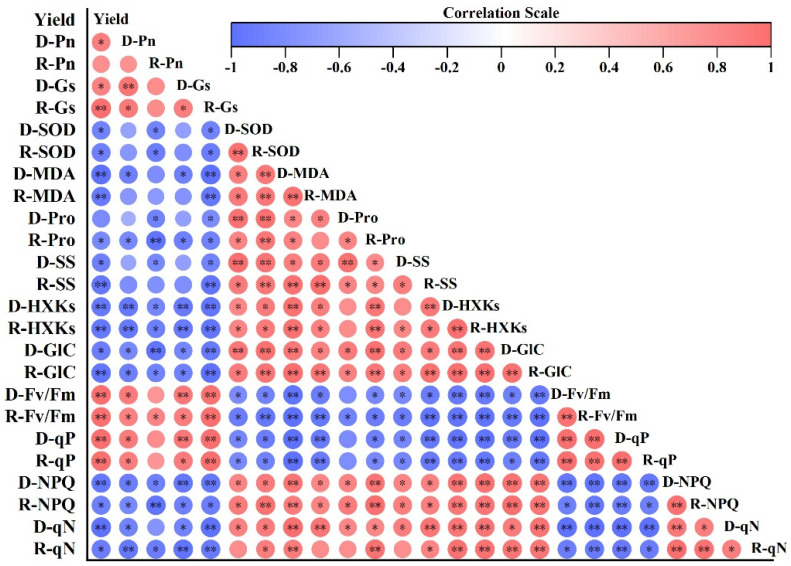
Correlation between the physiological indicators and yield. D, drought; R, re-watering. * *p* < 0.05. ** *p* < 0.01.

The DC value for each indicator was presented in the form of a box plot (Figure 4). After drought and re-watering, all of the indicators for both cultivars changed to varying degrees. The height of the box plot was inconsistent, indicating that the sensitivity of the indicators to drought stress and the degree of recovery after re-watering were different. The correlation between physiological indicators and yield was further analyzed, and a correlation was identified among those indicators. Most correlations were at a significant level. As a result, the overlapping of the information characterized by these indicators (Figure 5) could not be used directly to assess the drought tolerance of wheat.

#### 3.5.2. Principal Component Analysis (PCA)

To thoroughly analyze and compensate for the deficiency of the drought tolerance evaluation for each indicator, it was necessary to further use PCA and other methods to comprehensively evaluate the roles of these indicators. Based on the PCA, the DC values of 12 individual indicators showed that the cumulative rate of contribution of the two factors reached 83.46%, and the characteristic root was > 0.78. The two factors were extracted, so that the original individual indicators could be converted into two new independent comprehensive indicators, denoted by F1 and F2, respectively (Table 3). F1 had higher loadings on the fluorescence parameters (Fv/Fm, qN, NPQ, and qP) and osmotic regulatory substances (SS, Pro), while F2 had higher loadings on Pn, SOD, MDA, and GLC. 

### 3.6. Screening of Drought Tolerance Indicators

#### 3.6.1. Grey Relational Analysis 

**Figure 6:**
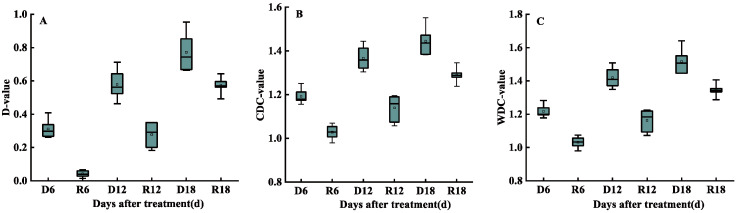
Changes in the comprehensive evaluation values of drought tolerance. (**A**) Drought tolerance comprehensive evaluation (D); (**B**) comprehensive drought tolerance coefficient (CDC); and (**C**) weight drought tolerance coefficients (WDC) under drought re-watering conditions. D, drought; R, re-watering.

The D, CDC, and WDC values for each indicator were obtained using Equations (2), (5), and (7), and presented in the form of a box plot. With the increase in stress duration, the D, CDC, and WDC values increased, and they decreased to a varying extent after re-watering conditions were applied (Figure 6). The degrees of correlation between the DC and D values for each index were ranked as SS, MDA, Pro, GLC, SOD, NPQ, qN, HXKs, Pn, Fv/Fm, qP, and G_s_. This reflects the closeness of DC and D values for each index, which was basically consistent with the sensitivity of each index in response to drought stress. In addition, the degree of correlation between the DC and WDC values of each index was ranked as qN, NPQ, SOD, GLC, HXKs, Pro, MDA, Fv/Fm, qP, Pn, SS, and G_s_, which largely coincided with the closeness of the DC and D values for each index (Table 4).

#### 3.6.2. Construction of a Regression Model

A stepwise regression analysis was performed, where the DC values of each indicator were kept as independent variables, and the D, CDC, and WDC values were used as dependent variables. The decisive coefficient (R^2^) of the three regression equations obtained was approximated to 1, and the F−test reached a significant level (Table 5), indicating that the three models fit well and were highly accurate at making predictions. All of them can be applied to the evaluation of wheat drought tolerance at the booting stage. Among all three models, the least number of indicators matched with the drought tolerance evaluation model constructed with the D and DC values, which were G_s_, Pro, MDA, SS, HXKs, GLC, and NPQ, and these indicators also existed in the other two models. This indicated that these seven indicators could be used as key indicators to evaluate the drought tolerance of wheat at the booting stage.

## 4. Discussion

### 4.1. Physiological Responses of Wheat to Different Durations of Drought

Similarly to other environmental stresses, drought affects many physiological and biochemical processes within plants. One of the most detrimental consequences is its negative impact on the photosynthetic rate [30]. As a result of water stress, the stomata in leaves close during the early stage of drought, which consequently reduces the uptake of CO_2_ to below the amount that is required for photosynthesis. This disrupts photosynthesis, and can lead to an imbalance between the photochemical activity of PSII and the electrons required for the Calvin–Benson cycle, which results in the excess absorption of excitation energy and subsequent photoinhibition damage to the PSII reaction centers [31], and ultimately leads to a decrease in crop photosynthesis [32]. Numerous studies have shown that severe drought stress can significantly reduce the photosynthetic capacity of crops, which causes a decrease in the chlorophyll content and net saturated photosynthetic rate (Asat), G_s_, and Fv/Fm [33,34,35,36]. However, drought stress leads to an increase in NPQ, which is an extremely important photoprotection mechanism in plants that helps them to maintain photosynthesis and consume light energy absorbed in PSII of leaves in the form of thermal energy [37]. This ensures that the plants grow and absorb CO_2_ under water deficit conditions [38,39]. This study showed that the values of Pn and G_s_ for both cultivars significantly decreased under drought stress (Figure 2A,B). The PSII activity was significantly reduced under prolonged drought with a decrease in the Fv/Fm and qP values. In contrast, the qN and NPQ values increased (Figure 2C–F), which coincides with the results of previous studies. These results indicate that plants close their stomata in response to stress, possibly to reduce water loss through transpiration as a damage control strategy.

Drought stress reduces the uptake of CO_2_ and subsequently reduces carbon fixation, saturating the electron transport system and forming ROS which, in turn, promote oxidative stress and lipid oxidation [26,40]. As the first enzyme in the antioxidant system, SOD plays a crucial role in preventing oxidative damage [35]. Our results showed that, compared with the control, the activity of SOD increased significantly under stress conditions, and more SOD accumulated in the drought−tolerant cultivar (LH 22) than in the sensitive cultivar (ZM 366). The MDA, a key lipid peroxidation product [41], is an indicator of oxidative damage in stressed plant cell membranes [42]. Our data demonstrated that MDA rapidly accumulated under drought conditions, with significantly higher quantities observed in the drought−sensitive cultivar ZM 366 compared with the drought−tolerant cultivar LH 22. The drought−tolerant cultivars exhibited higher activities of the antioxidant enzyme SOD and a lower accumulation of MDA, which are consistent with the findings of previous studies [5,30]. Under moderate and prolonged drought conditions, the maintenance of cell expansion is regulated through osmoregulation, which reduces the water potential and ensures water uptake in many species, accompanied by an increase in Pro and SS [17,30]. Our results showed that Pro and SS increased significantly with the increase in stress duration, and the values were found to be significantly higher in LH 22 than ZM 366 (Figure 3C,D). SS can improve the tolerance of wheat to stress by improving osmoregulation, ROS detoxification, protein stabilization, providing cell membrane protection, such as Pro, and accumulating SS, all of which were significantly correlated with drought intensity [16,43,44]. As the major byproducts of photosynthesis, the carbohydrates such as sucrose, glucose, and fructose accumulate in the leaves during drought periods, and are important for many physiological and developmental processes in plants [45]. Our study suggests that the amounts of GLC increased significantly under drought stress (Figure 3F), indicating that sugar metabolites could boost the drought tolerance of plants. Under adverse conditions, they act as osmoprotectants and osmoregulators, which have been observed to significantly and positively correlate with the ability of many plants to resist drought [46,47], and was also evident in our observations (Figure 5). Additionally, GLC needs to be phosphorylated for further metabolism to provide energy to the plants. HXKs are bifunctional enzymes that are involved in both carbohydrate metabolism and sugar signaling, yet they are the only enzyme that phosphorylates GLC in plants [48]. Studies have shown that plants use HXKs as GLC receptors to interconnect nutrient, light, and hormone signaling networks and thus, regulate plant growth and development [49,50]. Plomion et al. [51] found that there was a decrease in the expression of glycolytic enzymes in response to drought stress, and this decrease was linked to a reduction in biochemical mechanisms and the formation of new tissues. Once water conditions are restored, the inhibition of glycolysis is a mechanism that has been observed to accumulate sugars as an energy source for recovery and rapid growth. Alternatively, glycolysis appears to increase under drought conditions to provide the energy needed to activate stress defenses, particularly when photosynthesis was inhibited [52,53]. 

### 4.2. Compensatory Effect of Re-Watering on Wheat Physiological Characteristics

The physiological properties of plant drought tolerance are expressed under both drought stress and the recovery process that follows re-watering. The inhibitory effect of drought on plant growth can be compensated by appropriate water supplementation [32]. The leaf water potential in maize and cotton has been reported to decrease under water deficit conditions, but it rapidly recovers to equal or higher levels after re-watering, as compared with the control, to compensate for the loss of growth owing to drought [54,55]. By re-watering, the wheat photosynthetic rate could be restored to levels similar to those of the control after short−term drought stress; however, after long−term drought stress conditions, it could only be restored to levels up to 80% of the control [56]. The results of this study show that the physiological and fluorescence parameters of both cultivars recovered to the control levels at R 6, in which the Pn and G_s_ of the two cultivars recovered to more than 94.26%, indicating that compensation occurred during the short period of drought stress. However, at R 12 and R 18, the Pn and G_s_ of LH 22 recovered to 59.40–64.93% and 34.70–76.94% of the control, respectively, and that of ZM 366 recovered to 58.24–60.75% and 30.86–74.79% of control, respectively. These results suggest that prolonged drought damages the reaction centers photosystem I (PSI) and photosystem II (PSII). This is consistent with the findings of previous studies [57]. After well−watered conditions were met, the drought−tolerant cultivar LH 22 recovered more than the drought−sensitive cultivar ZM 366. Abid et al. [16] reported that the drought−tolerant cultivars demonstrated a higher photosynthetic capacity during drought and recovered faster after re-watering, with no significant decrease in yield compared with the drought−sensitive cultivars. Our study showed that the yield of LH 22 and ZM 366 were still decreased at R 6, R 12, and R 18 compared with that of the control, by 18.46%, 30.86%, and 45.90%, and by 29.38%, 42.61%, and 54.69%, respectively. Thus, the drought−tolerant cultivar LH 22 had a stronger ability to recover when compared with ZM 366, resulting in a lower yield loss. Overall, only mild, moderate, or short−term drought stress favored the formation of plant regulatory responses to a water deficit [33]. Re-watering after a short period of drought stress can not only improve photosynthesis and the yield of crops, but is also effective in reducing the damage to plants caused by oxidative stress [58]. Plants with antioxidant defense systems can combat ROS generation to an extent. This strategy is a good defense under normal growth conditions [59]. In this study, the antioxidant capacity and production of osmotic regulatory substances recovered in different manners when the plants were re-watered after drought stress. The activity of SOD and the contents of MDA and Pro of both cultivars recovered to normal levels following re-watering after 6 days of drought, indicating that a steady−state level of ROS generation and scavenging rates were reached that minimized oxidative stress. However, the other physiological indices were higher than those of the control despite some restoration that occurred after 12 days of drought; this showed that the oxidative stress situation persisted under prolonged stress, and ROS were overproduced and could not be overcome by the antioxidant defense system of the plant. This is consistent with the findings of a previous study [13,16]. An increase in sugar content has been reported to inhibit photosynthesis and growth, while it can increase the photosynthetic potential at lower levels [60]. The results of this study show that the SS of two cultivars recovered to control levels at R 6, and with prolonged stress, the SS of LH 22 was still 39.34% and 165.38% higher than that of the control at R 12 and R 18, respectively, and that of ZM 366 was 26.94% and 132.11% higher than the control respectively. The rapid decrease in sugar levels also indicated that the breakdown of sugar after stress relief provided the plant with enough energy to repair their damaged tissues [16]. After re-watering, the plants responded differently to photosynthesis by eliminating the ROS and adjusting the osmotic pressure depending on the duration of stress; this indicates that the physiological responses of plants were reversible in the range of 6 to 12 days of drought. These findings can further be used to subsequently analyze and evaluate the indicators.

### 4.3. Screening and Identification of Physiological Indices for Drought Tolerance in Wheat

The drought tolerance of crops is a complex quantitative trait, and it is challenging to reflect the drought tolerance using a single trait index [21], which only reflects the sensitivity of a trait without comprehensively analyzing the performance of crops under drought stress [25]. Crop stress tolerance is a synthesis of multiple physiological indicators based on genetic and environmental interactions [61]. As a result of the large number of physiological indicators, the response of each indicator to stress varies, and not all of the physiological and biochemical indicators can effectively and accurately identify the stress tolerance of plants. The correlation results of the experiment conducted in this study showed that the indicators were affected by drought stress to varying degrees, but there were some correlations between multiple evaluation indicators (Figure 5). This led to an overlap in information about the responses of crops to adversity [20], which made it difficult to assess the objectivity and accuracy of the results related to the identification of drought tolerance. Therefore, it is important to screen out reasonable indicators, in order to identify crop stress tolerance. Most of the studies that have screened out wheat stress tolerance indicators also used a combination of other agronomic parameters. Another previous study used the membership function method to calculate the flag leaf area (FLA), tiller number per plant (TN), biomass per plant (BMPP), plant height (PH), uppermost internode length (UIL), spike length (SL), spikelet number (SN), grain number per spike (GNPS), biological yield per plant (BYPP), grain yield per plant (GYPP), thousand kernel weigh (TKW), and grain volume weight (GVW), as indicators to evaluate tolerance under drought stress [21,62,63]. The crop response to drought and compensation after re-watering can more accurately be reflected by changes in physiological and biochemical levels than by changes in morphological characteristics. Therefore, in this experiment, 12 physiological indicators related to wheat drought tolerance, including photosynthetic characteristics (Pn, G_s_, Fv/Fm, qP, NPQ, and qN), SOD, and MDA, as well as osmoregulatory substances (Pro, SS, HXKs, and GLC), were selected to further screen physiological key indicators to evaluate drought tolerance in wheat. Previous studies have screened out the evaluation indicators of stress tolerance by combining principal component analysis and regression analysis, and successfully achieved good results in the evaluation of drought tolerance indicators for wheat [64], sesame [65], maize, and cotton [24,25]. Only a few studies were found that screened physiological indicators based on multiple statistical methods that combined principal components, membership analysis, and stepwise regression analysis. There is a lack of a comprehensive evaluation index designed for plant physiological and biochemical responses under drought stress and re-watering, as well as the dimensionality reduction in key sensitivity factors and the correlation between physiological indicators. Therefore, in this study, principal component, grey relational degree, membership function, and stepwise regression analyses; comprehensive evaluation indicators, such as the D, CDC, and WDC values; and the DC value of individual indicators, were used to eliminate the differences caused by different indicator units. The original 12 individual indices were transformed into two new mutually independent composite indicators via principal component analysis, which explained 83.46% of the total variation in 12 physiological and biochemical indicators. F1 primarily provided the Fv/Fm, qN, NPQ, qP, SS, and Pro, while F2 primarily provided the Pn, SOD, MDA, and GLC as relevant indicators that were screened unilaterally by a principal component analysis. The corresponding membership function values were obtained based on the rate of contribution of the two comprehensive indicators; they were weighted according to the weight of each comprehensive indicator to obtain the D value for grey relational analysis, which indicated the closeness of DC value to the D value. the correlation between DC and WDC values for all of the indicators largely coincided. Thus, this increased the accuracy and comprehensiveness of the evaluation study. According to the three equations obtained by the stepwise regression analysis, the least number of indicators matched with the drought tolerance evaluation model that was constructed with the D and DC values; these indicators also existed in the other two models. Therefore, seven individual indicators were selected, which included G_s_, Pro, MDA, SS, HXKs, GLC, and NPQ. Based on previous studies, the role of plants in response to abiotic stresses is multifaceted, and many physiological and biochemical parameters have been used to assess the drought tolerance phenotype of wheat, including Pro, SS, and MDA [66]. In this study, more desirable indicators were obtained by potted drought re-watering experiments during the booting stage, but the indicators selected and the screening methods differed from those that were used in previous studies. Thus, the indicators obtained were different. To our knowledge, the activity of HXKs has not been reported in terms of indicators used to screen wheat for drought tolerance. It is involved in carbohydrate metabolism and sugar signaling as a bifunctional enzyme. In this experiment, it accumulated significantly as the duration of stress increased, and rapidly returned to the control level after re-watering, which indicates sensitivity to drought and re-watering conditions. These results were combined to finally screen seven indicators, including G_s_, Pro, MDA, SS, HXKs, GLC, and NPQ.

**Figure 7:**
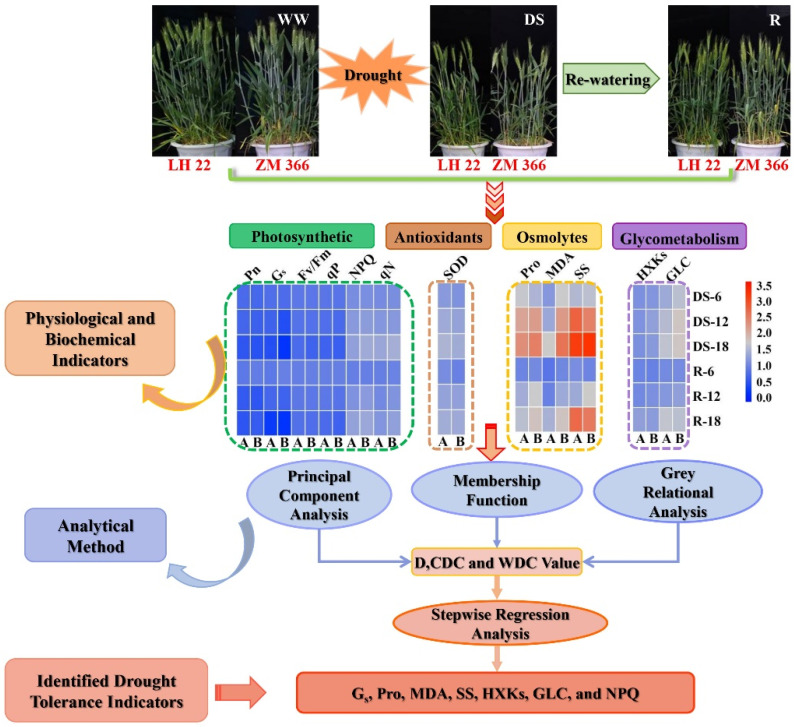
Schematic representation of wheat physiological and biochemical performance and drought tolerance indicators screening under drought and re-watering conditions. A: LH22 (drought−tolerant cultivar), B: ZM366 (drought−sensitive cultivar). WW: well−watered; DS: drought stress; and R 6, R 12, R 18: re-watering after 6, 12, and 18 days of stress treatment, respectively. D, drought tolerance comprehensive evaluation; DC, drought tolerance coefficient; WDC, weight drought tolerance coefficient.

We proposed a schematic model for the performance of physiological and biochemical characteristics, and the screening processes of drought tolerance indicators of wheat based on these findings (Figure 7), which can be used to identify drought tolerance of wheat at the booting stage.

## 5. Conclusions

The 12 physiological and biochemical indicators of the drought−tolerant cultivar LH 22 and the drought−sensitive cultivar ZM 366 that responded to different durations of drought (6, 12, and 18 days) and re-watering were evaluated at the booting stage in this experiment. Generally, LH 22 exhibited a higher photosynthetic capacity, antioxidant enzyme activity, and osmoregulation compared with ZM 366 under drought conditions, and the recovery level of LH 22 was also much higher than that of ZM 366 after re-watering. Finally, based on the methods of principal component analysis, membership function, grey relational degree, and multiple stepwise regression, seven indicators, including G_s_, Pro, MDA, SS, HXKs, GLC, and NPQ, were screened out as key indicators to evaluate drought tolerance in wheat. Since the G_s_ indicator can be easily and quickly obtained in the field, it can be used as the best drought tolerance screening indicator. In particular, G_s_ can be recovered to 97.30%, 74.82%, and 30.14% by re-watering when it decreased to 81.73–84.41%, 53.11–73.04%, and 28.85–54.00% under drought conditions, respectively. These findings can help to quickly and accurately identify the growth status of wheat after drought and re-watering, and evaluate its drought tolerance.

## Figures and Tables

**Table 1:** Effects of different treatments on grain yields of wheat.

Treatment	LH22 Yield (g pot^−1^)	ZM366 Yield (g pot^−1^)
WW	47.67 ± 0.70 a	51.80 ± 0.53 a
D	23.85 ±1.96 d	21.08 ± 1.59 d
R6	38.87 ± 1.65 b	36.58 ± 1.65 b
R12	32.96 ± 0.56 c	29.73 ± 0.45 c
R18	25.79 ± 1.00 d	23.47 ± 0.60 d

Data are presented as the mean ± SD. Different lowercase letters in the same column indicate significant differences between treatments at *p* < 0.05 (Duncan). WW: well−watered; D: drought (50 ± 5% relative soil water content was kept until harvest), R 6, R 12, R 18—re-watering after 6 d, 12 d and 18 d stress, respectively. SD, standard deviation.

**Table 2:** Combined ANOVA for physiological and biochemical indicators.

	Pn	G_s_	SOD	Pro	MDA	SS	HXKs	GLC	Fv/Fm	qP	NPQ	qN
Soil Moisture (M)	*	***	***	***	***	***	***	***	***	***	***	***
Cultivar (C)	ns	***	ns	*	***	ns	**	*	***	**	*	***
Stress Duration (SD)	***	***	***	***	***	***	***	***	***	***	***	***
M× C	*	**	ns	ns	***	ns	ns	*	**	ns	ns	ns
M × SD	***	***	ns	*	ns	**	**	**	*	ns	*	ns
C × SD	ns	**	ns	ns	*	ns	ns	ns	***	ns	ns	ns
M × C × SD	ns	ns	ns	ns	ns	*	ns	ns	**	ns	ns	ns

Note: analysis of variance (ANOVA), net photosynthetic rate (Pn), stomatal conductance (G_s_), superoxide dismutase (SOD), proline (Pro), malondialdehyde (MDA), soluble sugar (SS), hexokinase (HXKs), glucose content (GLC), PSII maximum photochemical quantum yield (Fv/Fm), coefficient of photochemical quenching (qP), non−photochemical quenching parameter (NPQ), and coefficient of non−photochemical quenching (qN). ns represents no significance at the *p* < 0.05 level. * *p* < 0.05. ** *p* < 0.01. *** *p* < 0.001.

**Table 3:** Eigenvectors and rates of contribution of the principal components for each index.

Index	Factor Loading	
	F1	F2
Pn	−0.808	0.418
G_s_	−0.892	0.088
SOD	0.809	−0.448
Pro	0.899	0.092
MDA	0.836	0.443
SS	0.937	0.015
HXKs	0.717	0.019
GLC	0.845	0.369
Fv/Fm	−0.959	−0.060
qP	−0.929	−0.105
NPQ	0.916	−0.202
qN	0.948	0.018
Characteristic root	9.235	0.780
Contribution rate (%)	76.957	6.504
Cumulative contribution rate (%)	76.957	83.461
Factor weight	0.922	0.078

Note: net photosynthetic rate (Pn), stomatal conductance (G_s_), superoxide dismutase (SOD), proline (Pro), malondialdehyde (MDA), soluble sugar (SS), hexokinase (HXKs), glucose content (GLC), PSII maximum photochemical quantum yield (Fv/Fm), coefficient of photochemical quenching (qP), non−photochemical quenching parameter (NPQ), and coefficient of non−photochemical quenching (qN).

**Table 4:** Correlation degree between the DC value of all the indices and the D value, together with the WDC value and the weights of their indices.

	Index	Correlation Degree with D Value	Rank	Weight	Correlation Degree with WDC Value	Rank
X_1_	P_n_	0.643	9	0.077	0.689	10
X_2_	G_s_	0.562	12	0.067	0.542	12
X_3_	SOD	0.707	5	0.085	0.877	3
X_4_	Pro	0.750	3	0.090	0.800	6
X_5_	MDA	0.767	2	0.092	0.743	7
X_6_	SS	0.828	1	0.099	0.679	11
X_7_	HXKs	0.692	8	0.083	0.851	5
X_8_	GLC	0.729	4	0.087	0.864	4
X_9_	Fv/Fm	0.642	10	0.077	0.726	8
X_10_	qP	0.630	11	0.075	0.694	9
X_11_	NPQ	0.704	6	0.084	0.879	2
X_12_	qN	0.704	7	0.084	0.917	1

Note: X_1_, Pn; X_2_, G_s_; X_3_, SOD; X_4_, Pro; X_5_, MDA; X_6_, SS; X_7_, HXKs; X_8_, GLC; X_9_, Fv/Fm; X_10_, qP; X_11_, NPQ; X_12_, qN. D, drought tolerance comprehensive evaluation; DC, drought tolerance coefficient; WDC, weight drought tolerance coefficient.

**Table 5:** Drought tolerance model prediction.

Dependent Variable	Multiple Stepwise Regression Equation	R^2^	*p*
D value	y = −0.492−0.148X_2_ + 0.136X_4_ + 0.108X_5_ + 0.105X_6_ + 0.076X_7_ + 0.091X_8_ + 0.135X_11_	0.999	0.0001 **
CDC value	y = 0.274 + 0.071X_1_ + 0.098X_2_ + 0.095X_4_ + 0.074X_5_ + 0.088X_6_ + 0.106X_7_ + 0.062X_8_ + 0.139X_11_	0.998	0.0001 **
WDC value	y = 0.333 + 0.098X_2_ + 0.093X_4_ + 0.087X_5_ + 0.108X_6_ + 0.107X_7_ + 0.069X_8_ + 0.107X_11_	0.998	0.0001 **

Note: X_1_, Pn; X_2_, G_s_; X_4_, Pro; X_5_, MDA; X_6_, SS; X_7_, HXKs; X_8_, GLC; X_11_, NPQ. CDC, comprehensive drought tolerance coefficient; D, drought tolerance comprehensive evaluation; WDC, weight drought tolerance coefficient; R^2^, decisive coefficient; ** significance at *p* < 0.01 by (F-test).

## Data Availability

Data are contained within the article.
